# Coronary endothelial dysfunction appears to be a manifestation of a systemic process: A report from the Women’s Ischemia Syndrome Evaluation – Coronary Vascular Dysfunction (WISE-CVD) study

**DOI:** 10.1371/journal.pone.0257184

**Published:** 2021-09-27

**Authors:** Sawan Jalnapurkar, Sofy Landes, Janet Wei, Puja K. Mehta, Chrisandra Shufelt, Margo Minissian, Carl J. Pepine, Eileen Handberg, Galen Cook-Wiens, George Sopko, C. Noel Bairey Merz

**Affiliations:** 1 Barbra Streisand Women’s Heart Center, Smidt Heart Institute, Cedars-Sinai Medical Center, Los Angeles, CA, United States of America; 2 Emory Clinical Cardiovascular Research Institute (ECCRI), Emory University School of Medicine, Atlanta, GA, United States of America; 3 Division of Cardiovascular Medicine, University of Florida, Gainesville, FL, United States of America; 4 Samuel Oschin Comprehensive Cancer Institute, Cedars-Sinai Medical Center, Los Angeles, CA, United States of America; 5 Division of Cardiovascular Diseases, National Heart, Lung, and Blood Institute, Bethesda, MD, United States of America; University of Bologna, ITALY

## Abstract

**Background:**

Coronary microvascular dysfunction (CMD) is prevalent in symptomatic women with ischemia but no obstructive coronary artery disease (INOCA). Urine albumin-creatinine ratio (UACR) is a measure of renal microvascular endothelial dysfunction. Both are predictors of adverse cardiovascular events. It is unknown if CMD could be a manifestation of a systemic process. We evaluated the relationship between renal microvascular dysfunction and CMD as measured by invasive coronary function testing (CFT).

**Methods and results:**

We measured urine albumin and creatinine to provide UACR in 152 women enrolled in the Women’s Ischemia Syndrome Evaluation–Coronary Vascular Dysfunction (WISE-CVD) study (2008–2015) with suspected INOCA who underwent CFT. Invasive CFT measures of endothelial and non-endothelial dependent coronary microvascular function were obtained. Subjects were divided into those with detectable (≥20 mg/g) and undetectable urine albumin (<20 mg/g). The group mean age was 54 ± 11 years, with a moderate cardiac risk factor burden including low diabetes prevalence, and a mean UACR of 12 ± 55 mg/g (range 9.5–322.7 mg/g). Overall, coronary endothelial-dependent variables (change in coronary blood flow and coronary diameter in response to cold pressor testing) had significant inverse correlations with log UACR (r = -0.17, p = 0.05; r = -0.18, p = 0.03, respectively).

**Conclusions:**

Among women with INOCA and relatively low risk factor including diabetes burden, renal microvascular dysfunction, measured by UACR, is related to coronary endothelial-dependent CMD. These results suggest that coronary endothelial-dependent function may be a manifestation of a systemic process. Enhancing efferent arteriolar vasodilatation in both coronary endothelial-dependent function and renal microvascular dysfunction pose potential targets for investigation and treatment.

**Clinical trial registration:**

https://clinicaltrials.gov/ct2/show/NCT00832702.

## Introduction

Prior work has determined that coronary microvascular dysfunction (CMD) is prevalent, associated with adverse clinical outcomes, poor quality of life and healthcare costs rivaling obstructive coronary disease in women with suspected ischemia with no obstructive CAD (INOCA)^1^. There are 3–4 million US patients with CMD, 100,000 new cases projected annually, and progression to heart failure with preserved ejection fraction (HFpEF) is most common, which is also more prevalent in women [1]. Further work suggests that CMD may be a manifestation of a systemic process. We have documented links between CMD and chronic kidney disease [2], and retinal microvascular dysfunction [3] in WISE women. Others have documented sex differences in retinal vascular [4] in hypertensive subjects, and brain small vessel changes in dementia [5] that are more adverse for women. Additionally, cross-sectional studies demonstrate a correlation between retinal microvascular changes and dementia, cognitive impairment, and brain imaging abnormalities [6]. These findings suggest the hypothesis that the rising burden of HFpEF and dementia that more often impact women may be due to a systemic microvascular dysfunction state.

Elevated urine albumin-creatinine ratio (UACR) is a measure of renal microvascular dysfunction [7], and has been suggested to be a marker of endothelial dysfunction [8, 9]. It has been studied extensively in patients with diabetes mellitus and is now recognized as an independent predictor of ischemic heart disease in asymptomatic as well symptomatic individuals without diabetes [10, 11]. Additionally UACR is an independent risk factor for clinical cardiovascular events including myocardial infarction (MI), stroke, cardiovascular death, and hospitalization for heart failure [12]. Furthermore younger, non diabetic and non hypertensive individuals also has shown correlation between UACR and subclinical vascular damage leading to cardiovascular diseases and death [13]. We investigated relations between two measures of microvascular dysfunction, CMD and UACR, to test the hypothesis that CMD is manifestation of a systemic process in women with suspected INOCA in the WISE—Coronary Vascular Dysfunction (CVD) Study.

## Methods

### Patient selection and procedures

**Fig 1:**
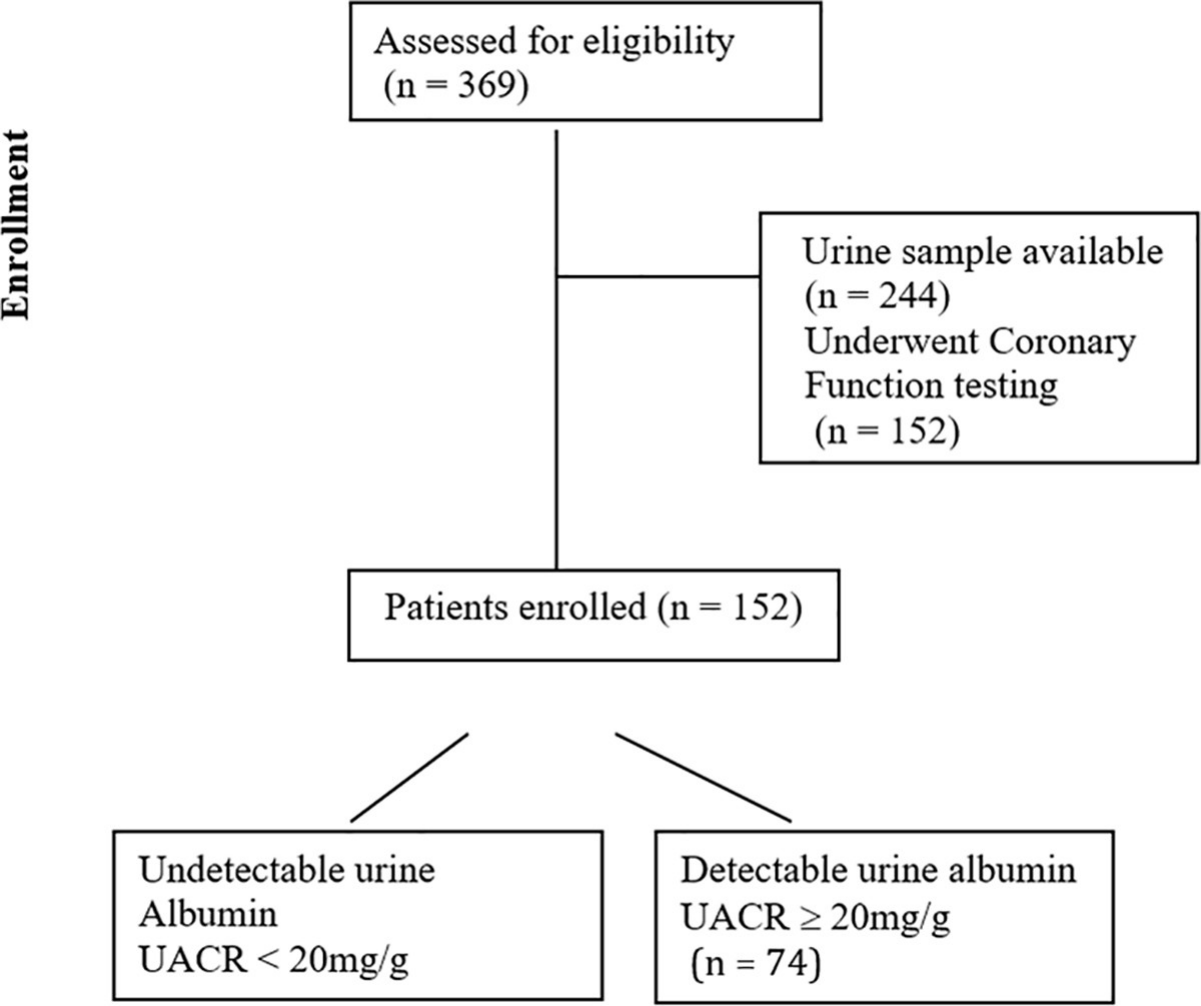
Flow chart for UACR CMD (WISE–CVD study).

The WISE-CVD study methods have been previously published [14]. Among the 369 women enrolled in WISE-CVD between 2008–2015, 244 had urine collected and measured for albumin and urine creatinine, and of those 152 (62%) had invasive coronary function testing (CFT) performed (Fig 1) and thus were included in the current study, as previously published [15]. Briefly, a doppler flow wire (FloWire® Volcano) was advanced through the diagnostic catheter and positioned in the proximal left anterior descending coronary artery. Intracoronary (IC) vasodilators and following parameters measured in response to IC adenosine (coronary flow reserve CFR normal ≥ 2.5), change in coronary blood flow in response to IC acetylcholine (ACH) (ΔCBF normal >50%), change in coronary diameter in response to acetylcholine (ΔACH normal > 5%), change in coronary diameter in response to cold pressor test (ΔCOP), and change in coronary diameter in response to nitroglycerin (ΔNTG) [15]. Institutional review boards at Cedars- Sinai Medical Center and the University of Florida, Gainesville each site approved the protocol, written and verbal informed consent was obtained from each subject, and data were monitored by an independent data safety monitoring committee.

Urine albumin (mg/g) and urine creatinine (g/dL) were measured via random spot urine samples using a commercial assay (Beckman Coulter Image® nephelometry). UACR was reported in mg/g, and in subjects with urine albumin in an undetectable range (<20 mg/g) UACR was reported as 0 mg/g. CFT was conducted as previously described [15]. Additionally, non-invasive cold pressor testing, an ice pack placed to the forehead (N = 75) or the left forearm (N = 77) for 2 minutes, was used as a non-pharmacological method for the evaluation of endothelial-dependent coronary microvascular function.

### Statistical analysis

Pertinent baseline characteristics were tabulated and are reported. Continuous variables were summarized as mean ±SD. Categorical variables are presented as percentages. UACR was skewed so a log transformation was used for all analyses. Spearman correlations were used to evaluate associations of UACR and the CMD parameters, as well as subgroup relations (n = 138–152). The cohort was also divided into those with undetectable urine albumin (<20 mg/g) and detectable urine albumin (≥20 mg/g) based on the sex specific cut off point as shown in prior studies [16, 17]. Results that used the entire cohort and incorporated UACR used a value of 10 mg/g for those with undetectable levels. Fisher’s exact test, student t test, and Wilcoxon rank sum test were performed as appropriate to evaluate for differences in baseline characteristics and CMD parameters between the two groups. Linear regression models included univariate predictors or variables of interest and used the outcomes of change in CFR, CBF, ACH, NTG, and COP adjusted for the explanatory variables of log UACR, systolic blood pressure (SBP), low density lipoprotein (LDL), body mass index (BMI), and indicators of whether the patient had statin or angiotensin converting enzyme inhibitor (ACE-I) or angiotensin renin blocker (ARB) use at baseline. The models were assessed mainly by analysis of residuals and comparing the change in parameter estimates when the subjects with undetectable UACR were added or removed. A Holm-Bonferroni adjustment was made to the p-values of the correlations as a group, and to the p-values from the regression model as another group [18]. A p-value of <0.05 was considered statistically significant for all analyses.

## Results

Pertinent baseline characteristics are summarized in Table 1. Most subjects had a moderate level of cardiac risk factors, with a low prevalence of diabetes. Mean UACR was 24.66 ± 78.45 mg/g among those with detectable levels. Overall, 78/152 (51%) had urine albumin levels <20 mg/g, the lowest detectable range. To better understand the relationship between CMD and UACR, subjects were divided into two groups, those with detectable and undetectable urine albumin. Baseline characteristics and CMD parameters were compared between the group with detectable urine albumin and undetectable urine albumin. Those with and without detectable urine albumin had similar rates of self-reported diabetes (13% vs 8%, p = 0.42), hypertension (43% vs 31%, p = 0.17) and similar systolic blood pressure (Table 1). With regard to the invasive measures of CMD, those with detectable urine albumin had a statistically significantly lower ΔCBF (52.4±65.7 vs 86.3±101.3, p = 0.04) but no significant difference in the other invasive measures of CMD.

**Table 1:** Baseline characteristics.

Characteristic (mean SD or %)	All subjects N = 152	UACR <20 mg/g, N = 78	UACR ≥20 mg/g, N = 74	p-value
**Age (years)**	54±11	54±11	53±11	0.861
**BMI**	30±8	30±8	31±9	0.705
**Caucasian**	72%	75%	69%	0.466**
**Heart rate (bpm)**	70±11	67±9	73±12	**0.0008**
**SBP (mmHg)**	126±18	124±17	129±19	0.106
**DBP (mm Hg)**	69±12	70±11	69±13	0.560
**Dyslipidemia**	12%	12%	12%	1.0**
**NT-pro BNP**	108.6±160.0	112.1±174.4	104.6±143.4	0.385*
**GFR**	91.7±17.6	90.3±16.7	93.1±18.5	0.342
**Hemoglobin (g/dl)**	13±2	13±3	13±1	0.309*
**Serum creatinine (mg/dl)**	0.8±0.2	0.8±0.2	0.7±0.1	0.235*
**UACR (mg/g)**	11.8±55.2, 0.1 (0.1, 576.2)	(undetectable) 0.1±0	24.1±77.5, 5 (1.1, 576.2)	By design
**CFR**	2.7±0.6	2.7±0.7	2.7±0.6	0.562
**ΔCBF (%)**	70.4±87.7	86.3±101.3	52.4±65.7	0.039*
**ΔACH (%)**	-7±19.3	-4.8±18.6	-9.3±19.9	0.157
**ΔCOP (%)**	-3.4±18.6	-0.9±16.1	-6.1±20.8	0.093
**ΔNTG (%)**	7.8±20.1	9.7±18.5	5.7±21.5	0.221
**History of Tobacco use**	39%	45%	33%	0.176**
**Diabetes**	10%	8%	13%	0.417**
**History of Hypertension**	37%	31%	43%	0.166**
**Family history of CAD**	47%	45%	49%	0.617**
**Statins**	39%	33%	44%	0.232**
**ACE-I/ARB**	22%	20%	24%	0.554**
**Full dose aspirin**	12%	9%	15%	0.314**
**Low dose aspirin**	55%	58%	51%	0.409**

ΔACH = change in coronary diameter in response to intracoronary acetylcholine, BMI = body mass index, NT-pro BNP = brain natriuretic peptide, GFR = glomerular filtration rate, CAD = coronary artery disease, ΔCBF = change in coronary blood flow in response to intracoronary acetylcholine, CFR = coronary flow reserve in response to intracoronary adenosine, ΔCOP = change in coronary diameter in response to cold pressor test, DBP = diastolic blood pressure, HDL = high density lipoprotein, LDL = low density lipoprotein, LVEDP = left ventricular end diastolic pressure, ΔNTG = change in coronary diameter in response to intracoronary nitroglycerin, SBP = systolic blood pressure, UACR = urinary albumin creatinine ratio Test p-values were from t tests except

*indicates Wilcoxon rank sum test

**indicates Fisher’s exact test.

Overall, endothelial-dependent variables (change in coronary blood flow and coronary diameter in response to cold pressor testing) had inverse correlations with log UACR (Table 2), such that heavier proteinuria correlated with more abnormal coronary endothelial function. There were no significant correlations between UACR and non-endothelial coronary function. Despite an overall correlation between ΔCBF and ΔACH (r = 0.46, n = 138, p<0.0001) there was no significant relationship between UACR and ΔACH (r = -0.14, n = 152, p = 0.09) (Table 2) when the entire cohort was examined and used a value of 10 mg/g for those with undetectable levels.

**Table 2:** Spearman correlations between CMD variables and UACR.

Variable	Correlation Coefficient	P-value	Holm-Bonferroni adjusted p-value
CFR, N = 144	0.025	0.770	1.0
among CFR ≥ 2.5 (normal, N = 89)	-0.007	0.947	1.0
among CFR < 2.5 (abnormal, N = 55)	0.134	0.330	1.0
ΔCBF, N = 138	-0.171	**0.046**	**0.598**
among ΔCBF ≥ 50% (normal, N = 69)	-0.20	0.100	0.910
among ΔCBF < 50% (abnormal, N = 69)	-0.043	0.728	1.0
ΔACH, N = 152	-0.137	0.091	0.910
among ΔACH > 0 (Normal, N = 64)	-0.017	0.895	1.0
among ΔACH ≤ 0% (Abnormal, N = 88)	-0.258	**0.015**	**0.225**
ΔCOP, N = 146	-0.18	**0.029**	**0.406**
among ΔCOP > 0% (Normal, N = 69)	-0.033	0.785	1.0
among ΔCOP ≤ 0% (Abnormal, N = 77)	-0.204	0.075	0.825
ΔNTG, N = 151	-0.132	0.107	0.910
among ΔNTG > 20% (Normal, N = 45)	0.024	0.876	1.0
among ΔNTG ≤ 20% (Abnormal, N = 106)	-0.179	0.066	0.792

Abbreviations as prior.

Among those with detectable UACR (n = 74) there were no significant correlations between UACR and other patient variables, including age, weight, BMI, N-terminal pro-brain natriuretic peptide (NT-pro-BNP) or angina measured by the Seattle Angina Questionnaire (SAQ), however we observed a positive and significant correlation between log UACR and LVEDP (0.26, n = 69, p = 0.03). In multivariable regression modeling that included all 86 subjects with complete ΔCBF, UACR, SBP, LDL, ACEi/ARB, statin use and BMI data, all variables except ACEi/ARB were associated with ΔCBF, so ACEi/ARB was removed from the model. (Table 3). If only those 39 with detectable UACR are included in the model the associations are smaller and not significant. In regression models with ΔACH, ΔCOP, ΔNTG, and ΔCFR as outcomes, UACR did not contribute significantly to any model, however creatinine was a significant independent predictor of CFR (parameter estimate 0.83 per 1 unit creatinine, p = 0.0409).

**Table 3:** Multivariable regression independent predictors of ΔCBF among those with detectable UACR (N = 86).

Variable	Estimate (SE)	P-value	Holm-Bonferroni adjusted p-value
UACR	-11.53 (4.55)	0.013	0.052
SBP	-1.23 (0.54)	0.025	0.075
LDL	0.93 (0.29)	0.002	0.010
BMI	-2.41 (1.18)	0.044	0.088
Statin use	30.17 (19.05)	0.117	0.117

Abbreviations as prior.

## Discussion

Urine albumin-creatinine ratio (UACR) as a measure of renal microvascular endothelial dysfunction has not been previously evaluated in our novel population of women with suspected INOCA [19]. Mohandas et al in prior study showed that reduced renal dysfunction is associated with CMD using eGFR instead of UACR [2]. In addition prior studies, Sakamoto et al and Chade et al provided similar association between CFR and CKD however using eGFR in patients with underlying CKD, unlike our patient population [20, 21]. Our results provide evidence that renal endothelial dysfunction, as measured by UACR, has a direct relationship to coronary endothelial-dependent microvascular dysfunction, whereby greater renal proteinuria correlated with more abnormal endothelial function. Conversely UACR did not relate to non-endothelial coronary measures, however our results may also involve varying degrees of epicardial vasodilation/mild vasoconstriction throughout the coronary tree, and therefore may not be specific to the microcirculation. Multivariable regression modeling demonstrated that UACR was predictive of ΔCBF and was the second strongest predictor after LDL-cholesterol. These results support the hypothesis that coronary endothelial-dependent microvascular dysfunction may be a manifestation of a systemic process.

Prior studies examined the relationship between renal function and CMD and found it as a marker of CMD, when estimated glomerular filtration rate (eGFR) was reduced [22], however, there has been no studies identifying UACR with coronary endothelial-dependent microvascular dysfunction. The association between microalbuminuria and CMD has been hypothesized to be due to a shared pathogenic mechanism of subclinical damage of the vascular beds in both the renal and coronary arteries [23], Ludic et al studied the relationship between CKD and coronary endothelial dysfunction in diabetics vs non diabetic patients, and found coronary endothelial dysfunction correlated with renal dysfunction in type II diabetic patients [24]. Prior WISE study demonstrated that renal function (estimated GFR [eGFR]), was significantly correlated with CFR (*r* = 0.22, p = 0.002), which persisted even when adjusted for age, diabetes, hypertension, dyslipidemia, double product, BMI, severity of obstructive CAD, and hormone replacement therapy (p = 0.0003, model R^2^ = 0.18) [2]. Other work has indicated that eGFR and UACR are markers of chronic kidney disease [25]. A study of diabetic patients (56% men) with no obstructive CAD was also consistent with the current results, demonstrating that endothelium-dependent vasoreactivity, measured by coronary artery diameter response to cold pressor, correlated with urinary albumin excretion rate (*r* = −0.39, p = 0.0003) [26]. Notably, only 14% of our study subjects were diabetic and all were women.

Multiple studies document that CMD carries an increased risk of adverse cardiovascular events [27, 28]. Data from several studies has established that microalbuminuria, which is measured by UACR, is an independent risk factor for clinical cardiovascular events including future stroke, myocardial infarction, cardiovascular death, and hospitalization for congestive heart failure [12] and an independent predictor of ischemic heart disease [10]. Moreover, ACE inhibition with the ACE-I ramipril in diabetes decreased the both adverse cardiovascular and renal outcomes [29]. As both UACR and CMD predict clinical adverse cardiovascular events, these results combined with the current results that link the two directly in our cohort, suggest that UACR may have prognostic and treatment implications in our novel population.

A potential explanation for this relationship between CMD and UACR is common risk factors contributing to endothelial dysfunction in both the coronary and renal microvasculature, including hypertension [30] and dyslipidemia [31]. Prior work in a study of 1567 subjects demonstrated major cardiac risk factors as independent correlates of urinary albumin excretion rate and prevalence of microalbuminuria in non-diabetic middle-aged men and women [32], suggesting that endothelial dysfunction is systemic [33]. Previous studies have found an association between kidney disease and microvascular dysfunction in the central nervous system [34], retina [35], and peripheral circulations [36]. Accordingly, albuminuria may represent a marker of global microvascular dysfunction, which includes renal and coronary endothelial dysfunction. This hypothesis opens the door to investigate future targets for treatment, for example ACE or ARB inhibitor therapy, long known to slow down renal dysfunction in diabetics with microalbuminuria [37–39]. A prior WISE clinical trial demonstrated improved CMD with quinapril which correlated with reduction in anginal symptoms [40]. In the LIFE study, subjects with a UACR greater than the median value at baseline, who were able to decrease their UACR to less than the median value at 1 year via use of losartan, had a reduced risk for cardiovascular mortality, stroke, and myocardial infarction compared with patients who were not able to decrease their UACR [41]. These studies suggest potential investigative areas for reduction of adverse events in CMD, as well as possibly prevention of HFpEF and dementia in women.

### Limitations

There are several limitations in this study to consider. Our subjects, referred for clinically indicated coronary angiography, likely do not represent all women or patients not evaluated invasively, as evidenced by their modest risk factor profile. Analyses in higher risk populations such as obstructive CAD might demonstrate stronger findings. Our analysis is limited to 152 women, did not include reference control subjects, and only 74 (49%) had detectable urine albumin. Although we used multiple testing adjustments, our multiple correlation and linear regression models tested may over-estimate borderline relations–repeated analyses in larger sample sizes is needed. Urine albumin and urine creatinine were collected a single time at the first study visit; transient microalbuminuria can occur due to fever, recent exercise, elevation in blood pressure, infection, such that the amount of falsely increased urine albumin levels cannot be determined. Lastly, as mentioned this study was conducted in exclusively women, and therefore potentially not generalizable to men.

## Conclusions

Among women with INOCA, renal microvascular dysfunction, measured by UACR, is related to coronary endothelial-dependent function. These results suggest that CMD may be a manifestation of a systemic process. Further, because both UACR and coronary endothelial-dependent function predict clinical adverse cardiovascular events, these results may have prognostic and treatment implications. Enhancing efferent arteriolar vasodilatation in both coronary endothelial-dependent function and renal microvascular dysfunction pose potential targets for investigation and treatment.

## Supporting information

S1 Data Supporting information data set. (CSV)
